# Let’s agree to agree: The situational academic quality of the UK REF as consensual public knowledge

**DOI:** 10.1177/03063127231152915

**Published:** 2023-02-06

**Authors:** Sveta Milyaeva, Daniel Neyland

**Affiliations:** Bristol University, Bristol, UK

**Keywords:** peer review, academic quality, consensus, calibration, UK REF, assessment, evaluation

## Abstract

The Research Excellence Framework (REF) is a UK policy tool for distributing government funding and an important indicator of the academic status of a UK university. The legitimacy of the policy comes from peers’ consensus on what academic quality is. We are interested in how the REF enables this funding distribution by determining the academic quality of a broad array of different forms of research through a single peer-review process. As they search for academic quality that is contingent to a specific epistemology and requires more time than the REF allows, how do academics agree to agree, and within constraints of a given timeframe? Interviews with REF panellists and their accounts of the process lead us to suggest that the consensus is enacted by setting up a situation: the mechanics of the REF with its practices of benchmarking, scoring, calibrating, and normalizing. This situation sets the boundaries of reviewing and, in doing so, propels peers to shift from assessment contingent on epistemic commitments to evaluation on a single scale. We argue that this shift renders academic quality distinct from scientific or epistemic quality.

For all academic researchers in the UK the REF is ubiquitous, mundane, and needs no explanation. REF is an acronym for the Research Excellence Framework, which in previous iterations was the Research Assessment Exercise (RAE) and the Research Selection Exercise (RSE). Dating back to 1986, these assessments comprise the world’s longest established system for competitive distribution of government research funding (Jump, 2013). The significance of these assessments as a form of income has diminished since tuition fees were introduced in 1998 (and raised in 2006 and 2013), but universities’ performance in the REF continues to be one of the most important benchmarks indicating academic status.

The centrality of the REF to UK academic life has been addressed in academic research, with social scientists conceptualizing what the REF is and what it does.^1^ As a technology of governance in an audit society (Power, 1997), the REF is said to be conducted in the name of accountability and transparency (Sayer, 2015; Strathern, 2000, 2002), by shifting trust relations from personal to evidential (Huber, 2013). It is also viewed as a vehicle for the marketization of higher education in the UK. For example, the introduction of an impact component in 2014, where universities report on how they have made a difference to public life, is crucial to what Watermeyer (2019, p. 4) calls ‘competitive accountability’ – a requirement to account for academics’ input ‘that privileges not academic or intellectual but market-driven values’. The assessment is now seen as creating a ‘quasi-market’ in university research (Brown & Carasso, 2013) and, by allocating finite research funds selectively, puts competition at centre-stage in making academic status as important as other sources of value, such as research grants and tuition fees (Neyland et al., 2019).

The pressure to perform well in the REF is more than a concern for institutions’ positions in ratings and rankings. The rankings are performative – that is, they are ‘constitutive rather than simply reflective of what they measure’ (Espeland & Sauder, 2016, p. 4). Strathern (2002) has argued that research assessment establishes measures that quickly become targets through which academic practice is reoriented. To meet these targets, UK universities have invested in REF managers, experts, and consultants who collectively establish the practices universities ought to adopt in order to succeed. Such success is established through forms of valuation that create academic research rankings (Espeland, 2020). It is this practice of valuation on which we focus: How does the REF value diverse academic research? How does it determine the ultimate academic quality of a broad array of different forms of research through a single process?

In viewing the REF as an evaluative mechanism, we draw on the wide range of scholarship that can be assembled under the umbrella of ‘valuation studies’, which sees valuation ‘as a social practice’, with an emphasis on valuation as action (Antal et al., 2015; Helgesson & Muniesa, 2013, p. 4; Muniesa, 2011). Although REF valuations are, ultimately, of economic worth – how much research money each department gets as a result of their REF performance – they are nevertheless a distinct form of valuing, based on academic evaluation through peer review.

Here we do not aim to provide a fully comprehensive account of the peer-review mechanics of the REF. Rather, hoping to contribute to conversations on ‘academic lives and cultures’ (Sismondo, 2019), we focus on how academics differentiate between high and low research quality, ultimately establishing consensus on it. Academic peer reviewing has been a long-standing subject of study (e.g. Lamont, 2009; Sabaj Meruane et al., 2016), but one observation is particularly apt in the context of the REF: Peer review is a process that results in a decision that is made *collectively*. For a decision on the value of research to be accepted and recognized, or to be legitimate, consensus is essential. In the REF case, consensus on academic quality emerges from assessing the seemingly incommensurable, heterogeneous, and not-immediately-apparent qualities of academic research outputs across diverse disciplines, from STEM to social sciences to humanities. Even within seemingly conventional and uniform disciplinary boundaries, ‘the disunity of science’ (Galison & Stump, 1996) points to the diversity of epistemologies and ‘machineries of knowing’ (Knorr Cetina, 1999) within these established boundaries. How can the process of peer-review produce a consensually objective or epistemically neutral measure of academic quality? In an environment of epistemological diversity and disunity, how do academics agree to agree?

The question of consensus in multi-epistemological peer review has been addressed mainly in relation to its reliability (Derrick & Samuel, 2017) by identifying biases (Berg, 2001; Huutoniemi, 2012; Langfeldt, 2006; Lee et al., 2013; Roumbanis, 2017; Travis & Collins, 1991). However, by viewing REF consensus as an achievement that reconciles a variety of epistemologies between and within disciplines, we are interested in the process of its formation.

To agree on the excellence of research across academic disciplines and within a discipline is to exercise individual judgement and negotiate it collectively. Various, and at times clashing, epistemologies of academic research never prevent a consensus, argue Lamont (2009) and Mallard et al. (2009). Focussing on peer review decisions regarding research grants in social sciences and humanities in the US, they look at how ‘excellence’, ‘diversity’, and ‘fairness’ are negotiated in reviewing research proposals. They recognize a palpable contradiction: While academics’ view of ‘what defines excellence is contingent on the cultural environment in which they are located, … they are encouraged to step out of their normal milieus to assess quality as defined through absolute and decontextualized standards’ (Lamont, 2009, p. 9). How do they resolve conflict and manage to produce a judgement (and agree on it) outside of their own epistemological context? The argument put forward by Lamont (2009, pp. 133–134) is that academics ‘loosen this association’ by recognizing the standards of the applicant’s field, and commit to a form of ‘[m]ethodological pluralism [that] produces universalism, thus bolstering the legitimacy of collective evaluations … as opposed to a more rigid cognitive coherence’ imposed by formal criteria of evaluations introduced across disciplines.

Our interest in academic quality prompted us to investigate REF evaluations done by academics across social sciences, humanities and STEM disciplines. The picture that emerges from our fieldwork is different from that outlined by Lamont (2009) and Mallard et al. (2009). The REF panellists’ evaluations that we encountered are most certainly pragmatic (Boltanski & Thévenot, 2006) resolutions of the challenges highlighted by Lamont (2009) and Mallard et al. (2009), but academic quality is an achieved agreement resulting from negotiations based on formal rules. As Lamont observes in a different context, in their evaluation and subsequent negotiations, academics involved in REF peer review are not primarily guided by their own epistemological conventions. Nevertheless, their evaluations still rely on criteria to measure against. Instead of adopting the epistemological principles of the research in question, we observed that academic consensus emerges through negotiations formatted by new evaluative boundaries set up by the machinery of the REF. The REF procedures and criteria establish a specific situation, a set of circumstances, which resolves the conflict of multiple epistemologies, by providing a means for academics to step out of their respective epistemic cultures to define epistemologically neutral academic quality. In this REF situation, ‘stepping out’ involves shifting modes of judgement from *assessment* (relying on epistemologies that are ‘native’ to peer-reviewers) to *evaluation* (using REF criteria and procedures), ultimately forming a consensus ‘that makes [it] possible to keep together beings whose justification would entail their separation into different worlds subscribing to different orders of generality’ (Boltanski & Thévenot, 2006, p. 18). We are going against the grain here. Before 2014, the system was called the Research Assessment Exercise and academics still commonly referred to it as an ‘assessment’. We use the term ‘evaluation’ because it derives from ‘value’ as a signifier for economic domain.

Our argument is based on 34 semi-structured in-depth interviews with REF participants. Although formal minutes of panel meetings are published and offer insights into the evaluation machinery, there was no data available on how individual panel members make judgments or on their collective negotiation with their colleagues. To get first-hand accounts of reviewers’ experience, we emailed randomly-selected panellists, making sure that our sample represented disciplinary (and epistemic) diversity. We travelled across the UK and interviewed academics from across all four main REF panels (comprising 36 disciplinary subpanels), as well as managers and practitioners assessing impact, resulting in over 1000 pages of interview transcripts (see below for a more detailed introduction to the REF). In our conversations with panellists, we discussed their workload and time, their reflection on the REF guidance and panel composition, their accounts of scoring and calibration practices, and the joys and discomforts of their experiences of the REF. We anonymized the conversations with our interviewees, who generously and enthusiastically shared their time and thoughts on the process.

## How to fund fairly? The REF as a higher education policy tool

The REF ‘must take its place alongside death and taxes’, wrote Bekhradnia (1999), a civil servant who is viewed as one of the architects of the REF (Richards, 2001). Indeed, there is no more certainty in UK academic life than the certainty of the research evaluation. As a formal exercise, it takes place once every few years, but it never really ends, turning into an internal audit of research active staff as the department gears up for the next round of evaluation as soon as the previous round has ended. As a constitutive part of UK academia, the REF stimulates much commentary: It is here to stay, yet what it is and what it does is debatable. To unpack the production of a tangible outcome – a consensus on epistemologically neutral academic quality – we must first appreciate the various complexities and contestations that underpin the evaluation. A good place to start is the very purpose of the REF, the question of what the peer-review consensus on academic quality is for. Historically, evaluation grew from a need for allocation of resources:

[I]n 1991, when the polytechnics became universities, and the money didn’t increase, we had to find the way of allocating the money selectively, and the only rational way of doing that was on the basis of the quality of the research outputs. Essentially you then agree that there has to be research assessment. That’s why I said about death and taxes. As long as we need to allocate the money selectively you need some sort of research assessment. (RAE manager)

Prior to this, public financing of higher education institutions in the UK had been increasing steadily since the 1920s. However, the expansion of higher education that followed the 1988–1992 government reform, which made polytechnics into universities and resulted in an increase in participation in higher education, prompted a different approach to funding (Milyaeva & Neyland, 2020). Choices needed to be made on how funding should be distributed. Such selectivity required a form of evaluation, and the evaluation required a mechanism.

That mechanism existed, in ad hoc form, in the Research Selectivity Exercises that had been conducted in 1986 and 1989; from these, the more thoroughly designed machinery of the Research Assessment Exercise (RAE) arrived in 1992, and was subsequently tweaked through the 2001 and 2008 rounds (Jump, 2013). By the round of 2014, the exercise was renamed the Research Excellence Framework (REF), and the standard requirement was for each university department to submit their REF returns – a portfolio of research outputs (e.g., books, articles) produced by members of the department and selected through a round of internal assessments. Since their introduction in 1986, the exercises have used peer review as the most appropriate way of evaluating research: ‘We need a system which is transparent, based on academic judgements and gives us a basis for those judgements’ (Bekhradnia, in Richards, 2001). To peer-review the submitted research, the Higher Education Funding Council for England (HEFCE; acting on behalf of its counterparts in Scotland, Wales, and Northern Ireland) set up four main panels, each overseeing a broad academic area (e.g., natural sciences, social sciences, etc.) through thirty-six smaller disciplinary sub-panels, or ‘Units of Assessment’ (such as Clinical Medicine, Biological Sciences, Physics, Sociology, etc.). Sub-panel chairs were members of the Main Panels, and were responsible for allocating the submitted portfolios of research outputs to their sub-panel members based on their expertise. Usually, two reviewers were assigned to each output to score it on a scale of zero to four stars. The 2014 set-up included a requirement that departments submit an environmental statement (an outline of departmental research milieu such as grant income, number of PhD students, research centres), and a new element – non-academic impact case studies that provided evidence of the cultural, economic, social, policy, environment or health benefits of academic research carried out in the submitting department, and that were linked to academic outputs. An overall REF performance score for a department consisted of 65% derived from research outputs (e.g., journal articles and books assessed through peer review), 20% from impact and 15% from environment. The score became the basis on which the annual budget of up to £2 billion of ‘quality-related’ (QR) research funding was allocated among UK universities.

The described set-up is a device that implements the seemingly straightforward idea of selectivity in research funding. As articulated by a REF manager, a civil servant overseeing the 2014 round, ‘the need to have a policy like that derives from a scarcity of resource, and in order to implement that policy you need a way of measuring excellence … to ensure that every pound that you spend delivers the best value’ (REF manager). Yet this policy, the purpose of which would seem to be rather unambiguous – to identify and reward academic quality – was met by REF participants with various attitudes. REF panel chairs and panel members were acutely aware of this, with one panel chair summarizing that ‘not everybody played the same game’:

For some, the ranking was by far the most important thing. For others it was really important, I think, for the bigger universities especially, that they got significant amounts of funding because they have got many more mouths to feed. (panel chair)

The distribution of QR research funding annually depended on a calculation of the percentage of staff whose outputs were submitted for REF evaluation. Some departments submitted fewer members of staff to the REF to get a high ranking (based on a small number of people doing hopefully excellent research), fully aware that this would reduce their allocation of government money. These departments calculated that the amount they would lose in government QR funding was less than they would lose from other external sources (e.g., from tuition fees or grant applications where they could use their high ranking as a promotional tool) if their reputation were negatively influenced by a low score. Other departments sought to submit as many staff as possible in a strategy to try and boost their research power ranking (the percentage of staff submitted combined with the score of each member of staff) and their QR funding. Well-known, well-funded and well-established departments at some universities submitted most of their staff and expected to score highly on their outputs:

It is only a financial exercise. I never had illusions. So, when you do the REF for your university, it is the money that you’re looking at. You’re trying to maximize income. It’s nothing to do with academic quality. It’s entirely about the tactics to maximize income. The importance of REF in [the interviewee’s subject], of course, is complex, because student numbers are capped. And international, non-EU students are also capped, and fiercely capped. We are heavily fined if we exceed. So, no matter what our quality, it makes very little difference to recruitment in [the subject]. (panellist)

Although selectivity seems to serve the purpose of identifying and rewarding academic quality, selectivity is not understood and managed uniformly. It is contingent on what kind of result might prove favourable, who (and what kind of work) might contribute to such a result, and how this might be used to later narrate the success and strength of an academic department or a university. ‘Manager-academics’ making these decisions are seen as part of the neo-liberalization of UK universities (Loveday, 2018, 2021; Nash, 2019). This has been met with academic resistance (Sayer, 2015) and anxiety (Gill, 2014; Jump, 2015; Loveday, 2018). REF panel members, whose work is also submitted as REF outputs by their own departments, are all too aware of this, and the recognition is consequential for their own decision making:

With the pressure on staff to be doing teaching, research, enterprise, helping administer the university, is a big load. There are individual pressures on individual staff members of any discipline, actually. The ones who get writer’s block and just can’t get writing. This drives people to nervous breakdowns. These are serious … this whole assessment, metric assessment of things, is a major pressure that people, individually, feel. (panel chair)

REF can be used for performance management [i.e.] managing people out of institutions. So it is a process where you want to make sure that you are protecting the integrity of the exercise, but also protecting the disciplines, and making sure that REF cannot be misused to push individuals out of positions, or to allow Vice Chancellors to make decisions that can be blamed on REF, or can be attributed to REF. It’s a very delicate exercise. (panel chair)

Such careful navigation of these broader complexities of the evaluation is indicative of the REF panellists’ appreciation of the wider politics of the production of ‘academic quality’. It strips ‘academic quality’ of its simplicity, rendering it vexed. Managing the uncertainty is a question of managing the politics of evaluation, ultimately facing the challenge of how to reconcile two different modes of judgment belonging to two different ‘worlds’: the ‘market’ mode with its focus on rewarding success in competition, and the ‘civic’ mode that prioritizes the ‘pre-eminence of collectives, higher common principle’ of the academic profession (Boltanski & Thévenot, 2006, p. 185). This would seem to be a balancing act that is difficult already, a discontent that demands a compromise. In the next section we outline more challenges that REF panellists faced in identifying ‘academic quality’.

## How to judge? The REF and its discontents

### The time discontent

In November 2015, nearly a year after REF 2014 results were announced, the *Times Higher Education Supplement* published an account of a REF panellist titled ‘As the panel could not give a reliable view of quality, I had to resign’. The panellist argued that one of the reasons it was not possible to identify quality research was the volume of outputs each academic on a panel had to read:

In spite of everyone’s best efforts, the system does not constitute *peer review in any meaningful sense.* There is simply too much material to assess with the care that would be rightly expected for reviews for research grants, publications or promotions. I had to read about 75 books and 360 articles or chapters, as well as numerous other outputs, including cross-referrals from other panels. I was not given any leave from my institution, and although I spent most of spring and summer at my desk, I could often give only an hour or so to ‘reading’ books, and no more than 20 minutes to articles or chapters. Some colleagues had an even heavier assessment burden. (Anonymous, 2015, emphasis added)

REF panellists we interviewed expressed similar concerns; though they were cautious, so as not to undermine the quality of their own assessment work. As one interviewee put it:

We evaluated the amount of time that we had available, and we divided it by number of tasks that we had and worked out how much time we had, and realised that it was going to be less than the amount of time you would spend on refereeing if you were sent a paper from a journal to referee it. (panellist)

Though the process is a review by peers, the volume of work that needs to be assessed makes it difficult for the panellists to see and perform a conventional peer review – a process that demands much more time than was made available to the panellists. Discontent with the limited time for review highlights the conflict between different modes of judging, or valuing. If peer review in a ‘meaningful sense’ requires more time, how could a valid and reliable judgement be made in the short time that is available?

The challenge of assessing quickly while maintaining the rigour of peer-review arises from the incommensurability of these two modes of judgement. These assertions of incommensurability introduce a kind of ‘commensuration discontent’, which takes place when ‘different modes of valuing overlap and conflict [and] it threatens some cherished identity’, and mostly happens ‘at the borderlands *between* institutions’ (Espeland & Stevens, 1998, p. 332, emphasis added). On the contrary, Boltanski and Thévenot (2006) view these modes of judgement as ‘orders of worth’ that are not singular or exclusive to a specific domain: Different orders of worth (and justifications of what is good and just) could co-exist peacefully within a group or an institution. Indeed, although a ‘higher common principle’ and the importance of the epistemological integrity in knowledge production is a distinctly academic ‘order of worth’ that justifies good and fair quality in peer evaluations, competition, too, is integral to and ubiquitous in academic lives: It is the basis for how grants and jobs are obtained in highly competitive environments. As one of our panellist interviewees put it, ‘assessing outputs is what I do, it’s my job and I am doing it constantly – I am reading CVs on a daily basis, and assessing the quality of publications’. Claims of incommensurability or a ‘sense of injustice’ are triggered when ‘different orders of justice are confused, and especially when justifications of a market order are extended beyond their legitimate boundaries’ (Boltanski & Thévenot, 2006, p. 15). In the case of the REF, consequential decisions flow from a market logic that sees competition as a basis for selectively distributing research funding, with a standard and accepted academic practice – peer review – pushed forward as the basis for addressing any concerns about this logic. The time discontent emerges among the panellists when they are expected to perform peer review within a timeframe that does not accommodate conventional assessment of outputs.

### The disunity discontent

In designing the REF machinery, one of its engineers insisted that the evaluation ‘should be discipline-based [as it is a] way of identifying the good research where it really happens’ (REF manager). The assumed unity of a disciplinary community was seen as a guarantee that there would be a consensus on academic quality, too difficult to achieve across disciplines. Yet, when it comes to knowledge production and evaluation within a discipline, the reality and fundamental characteristic of science is the plurality and disunity of heterogeneous epistemic cultures (Galison & Stump, 1996; Knorr Cetina, 1999). Variety in epistemic cultures and epistemological debates makes any discipline an assemblage of disciplinary communities.

What is more, knowledge production machineries vary widely. On that account, agreeing to agree on *discipline-neutral* academic quality across 36 REF panels – in effect saying that four-star research in clinical medicine is commensurable with four-star research in drama – does indeed ‘seem like a minor miracle that consensus emerges from the sea of differences’ (Lamont, 2009, p. 52). How could the large set of heterogeneous, situation-specific, highly individualized qualities of academic research outputs be assessed across various disciplines? As one of our interviewees put it, ‘disciplines are different; the assumptions that are made about what counts or what’s significant are different in each area’ (panellist). Or, in the words of another interviewee: ‘If someone wanted to exhibit a building as an output, [how] would it be possible for it to be assessed on a fair basis alongside a journal article?’ (panellist)

Finally, but equally importantly, over the years the number of disciplinary ‘units of assessment’ has grown with each round – starting from 37 in 1986, and rising to 67 sub-panels (comprising 15 Main Panels) evaluating research in 2008 (Jump, 2013). The sector cost of the evaluation also grew, reaching £47 million in 2008. As the 2014 round of evaluation approached, HEFCE decided to merge some units into larger panels in the name of efficiency and cost savings (HEFCE, 2015).^2^ The cost savings were not achieved: The 2014 REF cost nearly £250M (Else, 2015). In striving to represent a discipline, REF disciplinary panels were disparate and intentionally so, because ‘how do we make sure that our concerns, and things that are very particular to us and our ways of working are represented?’ (panel chair). Ensuring diversity in each of the 36 sub-panels seemed crucial:

Initially, the members of the subpanel … were all nominated by the subject associations. One of the problems that I faced, because I, as Chair, had to look through this list of nominations and try and find a balance in terms of subject expertise, gender, kind of university, research approach, etc., etc. (panel chair)

But it was a fine balancing act as, once composed, sub-panels often proved difficult to hold together:

HEFCE continually wanted to limit the number of people. I ended up appointing more and more and more in order to make it work. They did eventually agree [but] it costs them money. But equally, keeping a panel together, I ended up having [N number of] people including the secretariat sitting around the table, that’s a very big panel to manage. To chair, to keep everybody together without it fractionalizing and so forth. … If you were to try to do this with somebody who was a manager who was not fully aware of the potential political and professional and other influences, there’s a real risk that the members would take advantage of them. Would sew everything up. Academics are very good at this. (panel chair)

What constituted impact (as a new category, evaluated by non-academics) was also much debated:

It’s a sort of unusual thing that the government came up with, really, just to say, ‘Somehow these [impact assessors] people are expected to come into this alien environment [of academia] and apply judgements which were based on a totally different set of criteria from anything they’d ever experienced before’? There was a sort of implication that users know what they’re using and why they’re using it … In my view that was the most difficult part of the whole REF process, was this sort of *meeting of quite distinct cultures* which thought they were both doing the same thing in some way. (panel chair, emphasis added)

Seen through this lens – the time pressure, the conflict in modes of judgment, the epistemological heterogeneity within and across the panels, the disunity in prioritizing what constitutes academic quality – all these challenges would seem to be insurmountable in reaching consensus on academic quality. A REF panel chair put it succinctly:

I’ve always had a view that, but I’m quite pragmatic about these things, it’s a really difficult thing to do. To say that you’re going to peer review all outputs from every single research that’s submitted in the UK. Do it in a particular time and get agreement. It’s actually very challenging. (panel chair)

Yet consensus was a priority. The REF operates as a policy tool by producing a single set of results that could not be appealed or amended and would then provide the basis for annually distributing money to university departments for up to seven years (with REF 2014 results being used to distribute funds up to 2021-22). Given the finality of the results, their legitimacy is essential, and consensus is instrumental to it.

### Disciplinary versus mechanical objectivity

To establish consensus, differences have to be reconciled, making evaluations compatible and acceptable to all involved. Consequently, the epistemological problem of how to objectively value research turns into a social problem of how to get everyone to agree. The legitimacy of the REF as a policy tool that selectively distributes research funds also required the perception of an objective position, distant from the intricacies of the academic world. The position of ‘mechanical’ objectivity of quantification as ‘knowledge based completely on explicit rules’ (Porter, 1995, p. 7) is what the REF was set up to achieve. A range of studies show the importance of distant objectivity, ‘the view from nowhere’ (Nagel, 1986) as a way to manage demands of public accountability (Collins & Pinch, 1993; Jasanoff, 1990, 2011, 2012).

The machinery of the REF was designed to produce a form of authority and trust that exercises judgments that are seemingly transparent, because they are based on formal criteria rather than esoteric, black-boxed forms of expertise where judgments are highly subjective, often based on tacit knowledge (Collins, 1985) with rules unknown to the public. Wynne’s (1988) term ‘white-boxing’ is apt here, as it shows that procedures based on explicit rules are employed to overcome the image of black-boxed expertise to demonstrate transparency and avoid doubt in qualitative judgements that are prone to bias and difficult to scrutinize. To achieve this trust and authority, Porter (1995, p. 229) argues, ‘a preoccupation with explicit, public forms of knowledge [is] most evident where knowledge is to be shaped for policy purposes [and it] does not depend too much on the particular individuals who author it’. Yet, quantified, transparent, objective judgements on academic quality in UK higher education had to be discipline-based, and this form of objectivity required the judgment of experts (academics carrying out peer review) that had to be shaped into a consensus.

As the panel chair attested above (and many panellists agreed), forming a consensus among epistemologically variously inclined experts is a difficult task. In its capacity as a ‘technology of inclusion’, commensuration makes ‘mechanical objectivity’ possible – it creates coherence and thus removes heterogeneity by submitting the variety of inputs to a single system of measurement. This, argue Espeland and Stevens (1998, p. 331), rebalances relations of power: ‘[Q]uantitative methodologies are the provinces of weak elites and [that is] why they are resisted by those whose authority depends on expert judgement, character, or informal knowledge’. To strike an agreement on academic quality, the REF evaluation required academics to shift between different ‘cultures of objectivity’ (Porter, 1995): ‘disciplinary’and ‘mechanical’, or from consensus within an epistemic community to a consensus between various epistemic communities. ‘Disciplinary’ objectivity (Megill, 1994; Porter, 1995), or ‘trained judgement’ (Daston & Galison, 2007) is a consensus achieved within an epistemic community.^3^ As Megill (1994, p. 5) argues, it is a ‘claim by practitioners of a particular … subdiscipline to have authoritative jurisdiction over its area of competence’. The time-consuming dedication to conventional forms of peer review outlined above reflects the postulate of an ‘anti-algorithmic and antimechanistic’ disciplinary objectivity, where ‘accuracy should not be sacrificed’ (Daston & Galison, 2007, pp. 331, 321). But it is precisely this that, the REF demanded, had to be replaced by traceable, transparent, and public mechanical objectivity. This type of objectivity is about quantification and rule-following, and ‘orientation away from [the] interpretive, … without that distortion characteristic of the observer’s personal tastes, commitments, or ambitions’ (Daston & Galison, 2007, p. 121). How was this shift ultimately accepted and enacted by experts?

## From assessment to evaluation: The REF as a situation

### Situational judgement

Recognizing the plurality of modes of judgements within a group, or an institution, suggest Boltanski and Thévenot (2006, p. 16), requires us to re-focus from the group to a situation. Faced with uncertainty, individuals, they write, ‘are obliged by the situation in which they are involved to shift from one mode of adjustment to another, from one measure of worth to another’. If various forms of worth are attached to a situation rather than to a collective, the situation ought to be in focus to understand how the shift between the forms of valuing, of establishing what is ‘objectively’ good, is enabled. To establish academic quality, the REF mechanics propel peers to shift from one ‘order of worth’ (their epistemic allegiance with its time-consuming assessment and its – often tacit and discretional – epistemological adherence) to another (based on consensus enabled by commensuration through explicit criteria). In the REF situation, the shift from private ‘disciplinary’ objectivity to public ‘mechanical’ objectivity is a shift from assessment to evaluation. In the REF context, the former deals with the question of research quality in relation to epistemic commitments, whereas the later adheres to criteria and locates quality on a given scale. Or, as one of the interviewees put it, ‘it’s a qualitative process that tries to apply a quantitative [measure]’ (panel chair).

The REF situation consists of a number of components enabling a shift from assessment to evaluation and, ultimately, achieves a consensus on academic quality despite the time and disunity discontent.^4^ This situation comprises the entire configuration of peer reviewing – the guidance, criteria, meeting set-ups, documentation, and technological infrastructure that facilitates the scoring, its recording and calibration. Put together, it is all of these elements that enable consensus, and collectively create the environment (the situation) that forces participants to evaluate rather than assess, to switch from disciplinary to mechanical objectivity. It rejects the wider epistemological incommensurability, the unmanageable ‘chaos of disciplines’ (Abbot, 2001) through what Scott (1998, p. 11) calls ‘a narrowing of vision’: ‘the great advantage of such tunnel vision is that it brings into sharp focus certain limited aspects of an otherwise far more complex and unwieldly reality [which is] more susceptible to careful measurement and calculation’. Indeed, a range of literature demonstrates the power of measuring and classifying tools as ‘instruments that absorb tension [and] stabilize a compromise’ (Boltanski & Thévenot, 2006, p. 279; see also Lampland & Star, 2009; Mallard, 1998), conceptualized as, for example, ‘objects for cooperation’ or ‘boundary objects’ (Bowker & Star, 1999; Bowker et al., 2015; Star & Griesemer, 1989). Or, as one of the panellists put it, ‘it’s overwhelming looking at it prospectively, but … having got a kind of *formula* for doing it [it] was okay’ (panellist).

### Ongoing calibration and relative scoring

The REF formula was a set of rules that were established and adjusted to pacify and homogenize differences in *assessing* by requiring *evaluation*. A measurement tool of a star scale (from one to four) was introduced to judge research quality. The tool was calibrated on an ongoing basis (before and during the evaluation stage of the exercise), its application was normalized, ultimately producing what we, following Mallard’s (1998)work on ‘metrological intercomparison’, call situational academic quality.

At the pre-evaluation stage of calibration, all four Main Panels (comprised of sub-panel chairs) jointly came up with an initial benchmark for evaluation: a set of criteria of ‘originality, significance and rigour’ for scoring outputs by all disciplinary panels. The aim was to ‘create universality’ (O’Connell, 1993) through inceptive instructions on what each number of stars on the scale means in these terms within and across the main panels:

Every time I went around the subpanels, the first thing you say is, ‘We’ve got to get the benchmarking process right. We’ve got to make sure that if somebody puts a four in a [discipline X] panel and a four in [discipline Y], that they have the same sort of currency’. You can’t have one very much higher or lower than the other. (panel chair)

[T]his time, each Main Panel had to have a virtually uniform set of rules. In one or two minor areas, there were some differences. But certainly, for the main panel, we just had one set of rules, effectively. … even across the main panels, again, they were trying to impose a much more uniform structure, making it less idiosyncratic. So, even what we were putting into our Main Panel, they would still have to then square that off with the other Main Panels, and there was a lot of uniformity. (panellist)

These rules of interpretation were also tested on a small set of outputs among the members of the Main Panels, and their scores were compared to see if the universalizing benchmark worked in a situation of high uncertainty and variance. As our interviewees explained, ‘you were being asked to calibrate papers from completely different areas that you didn’t know anything about’, and ‘there was such huge disparity [and] we needed to educate each other, to be open-minded about certain kinds of research’ (panellist interviews). It also got panellists ‘to think about how we are going to read this material and come to a decision on its quality’ (panel chair). This possibility of a different way of reading unfamiliar research was enabled by the process of calibration of the initial benchmark. Epistemic adherence that guides assessment (or conventional peer review) of a specific piece of research is about zooming out to capture as many epistemic possibilities as possible. In contrast, the evaluation mechanism of the REF was about zooming in on the outlined criteria, with the goal of coming up with ‘shared understanding across subpanels and main panels of what a one, a two, a three and a four [star research output] might be’ (panel chair). This ‘shared understanding’, or normative interpretation of the criteria aimed not at completely eliminating differences, but instead establishing universality and consensus through accommodating differences, at least to an extent: ‘there was a trading and reconsideration during that criteria setting’ (panel chair).

Yet, to what extent and in what ways were negotiated ‘shared understanding’ accepted in all 36 sub-panels? The trials of calibration continued in different sets of metrological arrangements – the suggested interpretive convention was taken to the sub-panels where the chairs conducted the same calibration exercise within their panels. Drawing on the results of the Main Panels’ calibration, they asked their sub-panellists to score a small range of outputs. As one of the sub-panellists reflected on this process: ‘[The panel chair] stuck with the guidelines but he told us where to look, and by the end I knew exactly where to look’ (panellist). Here is one of the panellists reflecting on the process of scoring:

Once you’ve done it, I certainly felt near the end that my little mental formula for scoring a paper was starting to become too formulaic. Early on I really struggled with how to score a paper, somewhere in the middle, at the end of the first one and the beginning of the second one I was doing it, I was reading the paper properly and thinking about it. Quite near the end I was feeling, ‘I know how to do it. Read the instructions. Look at the figures and give it a score’. … I can remember the new people going, ‘That’s impossible to read 700 papers’ and we all went, ‘No’. But it was that sort of thing, here’s how to do it. I think that was helpful as well. (panellist)

More intercomparison followed as the scores produced by different panellists were then compared and adjusted to establish an in-panel, local convention to use in their actual scoring to conciliate different ways of reading outputs. This was designed to reduce the impact of ‘the hard markers and the easy markers’ (panellist):

it was important to make sure that everybody used the same scale, because you know maybe I’m not generous and I will give a paper a three but a colleague will be more generous and give a paper a four, and *we don’t want it to depend on the panellists themselves*. … the main purpose of that was to make sure that there were no differences across panellists. (panellist, emphasis added)

Once all the outputs were submitted for scoring, usually two sub-panel members would score an output and when the scores were in the system, differences between scores were flagged up and settled, sometimes by inviting another panel member to look at the output in question. As one of the panellists put it,

at every stage in the process, the panellists who are doing the work on the panel can have what they are doing checked. The panels were micromanaged in a detailed way, by technical staff but also in a way by the main panel chairs … you’re doing everything in such fragmented manner, and you are being micromanaged as you do it. … The panels are no longer panels. They’re sub-panels. There are numbers of panels in a main panel, but the main panels are also higher up the food chain, and they’re micromanaged. … I think it was very different [in a previous exercise], where the chair and the panel as a whole had much more autonomy in how the panel worked. I think that the leeway and the ability to do things other than to enact the rules was much more circumscribed in the REF. (panellist)

Recognition of the contextual nature of scoring, with the numerical value attributed to one paper being set relative to the score applied to other papers involved, as Boltanski and Thévenot (2006, pp. 16–17) put it, ‘practical modes of reasoning … obliged by the situation’. We also might view calibration as agreement through a finitist lens (Barnes et al., 1996). There is an open-endedness to the 4-star scale, and thus the extension of the instances of the classification – e.g. is this a 2-star or 3-star article? – is on the basis of resemblance to already rated (or classified) article. These instances are then further negotiated through calibration, that is, by figuring out specific empirical characteristics of each category. One panellist explained:

I don’t know, it seems artificial, obviously, because what happens is, you come up with your own set of criteria, and you give your marks, and then you find they’re way out of kilter with others. Well, ours were out of kilter with other people’s, and we had to just make an across the board adjustment. … There was a certain point where I had to make, at some point, what might seem as reasonably arbitrary adjustments. But, in a sense, I don’t think that’s necessarily a bad thing. The fact is, the *numbers are* kind of meaningless, it is *relative; what’s important is the relative scoring, not the absolute numbers*, so if you have to adjust your numbers, that’s fine. (panellist interview, emphasis added).

Yet again, the de-individualized scores for the same output, whether easily agreed on or arrived at through reconciliation and adjustment, were still not fixed: They were, as Mallard (1998, p. 591) puts it, ‘floating over the collective’. The post-evaluation stage was about continuously re-adjusting and aligning the distribution of scores within and between the panels. The application of the initial normative benchmark (the rules of interpretation of the scale) demanded consistency between scores for a single output, but it also demanded normalization of all panels’ scores. This happened in a number of ways, depending on the location (a specific panel) of the stabilization. Here is one of the panellists explaining the way it was done by their panel:

We actually used automatic methods to align the histograms, the distributions produced by the different scorers. … It was a much better method, and everybody should use it, because it’s based upon this understanding that what we’ve got is individual scorers, each producing unaligned distributions, and actually *you need to normalize*. … We did do some calibration exercises [but] we knew, right from the outset, that we were going to do automated normalization, which is much more reliable. And not to do, ‘Do you think it’s a three? Do you think it’s a four?’ is just rubbish. So, achieving inter-rate consistency is almost impossible, so what you do is you make quite sure that your people are doing intra-rate consistency. And then post-hoc alignment. (panellist interview, emphasis added)

So, the de-individualization continues, and the re-adjustment expands, incorporating various local instances, and the initial benchmark of academic quality becomes more universal and stable:

[I]n our meetings, we would get feedback from our chair who’d been to the main panel chair, and [they] would say … ‘We’re doing okay, chaps. We’re on the right track’. So, that was going on the whole time, and we had a lot of pressure on us to get done by these deadlines. There was a lot of time-pressure for the deadlines, and specifically so they could do the calibration. (panellist)

Individual voices are fed into the overall results which become aligned and are made consistent through normalization. Abbot’s (2001) disciplinary ‘chaos’ and the time and disunity discontents are, overcome:

It’s easy to do a calibration exercise on one piece, one output, but when it’s then co-existing in a field of 400 outputs and there has to be something relative, everything that you’ve said about that one isolated output stops being relevant. … Eventually an algorithm was applied that brought grades into balance both within the panel and between panels. Such an *application of a computational model does not allow for the breadth and multiplicity of positions in a field to be recognized*. (panellist interview, emphasis added).

Stabilized interactively, in relation to all other instances of its interpretation and use, this normative consensus on what constitutes each of the four degrees of academic quality is produced by the REF situation, it is situational in the sense of being ‘conventionally true’ (Mallard, 1998).

### Academic quality as situational judgment

REF academic quality is an outcome of consensus built on absorbing tensions between different epistemics. The initial epistemic polysemy of what constitutes academic quality is, in the words of Dewey (1937, pp. 105–106), something that is doubtful. It is an indeterminate situation where elements of it ‘do not hang together’, where responses to the issue at stake are as conflicting as they are multiple and dissimilar. However, he argues, the doubtful does not belong to us, its resolution is not simply a matter of ‘“mental” processes’, it belongs to the situation. And this situation, the mechanics of the REF – with its practices of benchmarking, scoring, calibrating and normalizing – provides the means to switch from one order of worth to another, from the principle of assessment to the rules of evaluation, thus resolving the uncertainty of what constitutes academic quality in this situation. The existence and worth of two distinctive modes of judgement (two objectivities, adhering to either an epistemic assessment or an evaluating mechanics) is certainly acknowledged by panellists:

What we’ve found of this exercise that there was somebody who was scoring very low on everything. And we just had to kind of talk this person around and say ‘Look, you are not rejecting these papers [laughing], *you are not refereeing*, and if you mark down all the papers in our subjects because you know about them and you think they have small mistakes, then that’s not really the message you should be sending out’. (panellist interview, emphasis added)

But the vision of panellists is narrowed, zoomed in on REF terms:

[My] reviewing experience of working for [discipline X], when you get reviews back you see that some reviewers are very sectional. They will just say, ‘They have used this approach and that’s terrible, they should be using my approach instead’. I just think, for the [REF] process to be fair, then things should be judged on their own [REF] terms and not by people’s personal methodological or theoretical preferences. That is a challenge when you are ranking things, because you are aware of what you are doing, being the ranking of how well things are presented [in the REF terms] rather than the substantive content. (panellist)

Most significantly, acknowledgement of the two principles available, and the choice of using the REF terms as the terms of consensus, was not widely viewed as a trade off at the expense of disciplinary objectivity.

In a way, isn’t that what we do all the time? I’m not sure that it’s so distinct from the hat that you change when you’re reviewing a research grant, or even assessing a PhD or whatever. … I think that’s only an extension of what we do a lot of the time, even in our own work, isn’t it? We’re constantly reviewing and assessing. … That’s not quite the same thing as putting a mark on, a score on somebody and then giving them, effectively, government money, I know, but when you’re doing it, it just feels like you’re doing a normal part of academic reviewing. That’s just what it *feels* like. (panel chair interview, emphasis added)

It was a lot of work, but that’s how peer reviews have got to operate, so I didn’t think there was anything exceptionally problematic in doing that. I think that the task of the evaluating the pieces of published work according to certain criteria, even though … the exact specification of what was worthy of which kind of star ranking I found problematic, but the actual business of doing it, no, it was not too hard. (panellist)

The situational shift from disciplinary to mechanical objectivity without undermining either, or without implying ‘a relativism of values’ (Boltanski & Thévenot, 2006, p. 215) was distinctly summed up by a panel chair:

I was heavily criticized at one point on the panel where I made an intervention which I referred to assessing impact as a game, by which I meant there were a set of rules and as in a game, you play according to the rules. … And it was intended to be a provocative remark, but it was intended to provoke in a different sort of way. Whereas actually they thought, ‘No, we’re not just playing a game. We’re taking it seriously’. What, essentially, we were doing was an artificial exercise and I wanted people to remember that. … [T]he exercise itself, I have absolutely no doubt about the integrity of and I would stand by, as it were, the detail that we constructed on the basis of the exercise. (panel chair)

Such co-existence of both types of objectivity matters, if the end result – the consensus on academic quality – is to go beyond the scores and become a policy tool, accepted as a legitimate basis for reward.

## Conclusion

In all its incarnations, the REF has been met with fierce criticism across the UK higher education sector. UK academics, including our interviewees, lament gaming through recruitment strategies, fractional contracts, inflated academic reputations, self-promoting strategies, and increased publishing pressures as the REF’s most detrimental effects. Equally important, it is widely acknowledged that the REF is conductive to ‘goal displacement’ (Merton, 1968) where anticipation of the appraisal shapes the focus, type and methods of academic research. Yet what is not disputed is that the REF is a collective mechanism for producing consensus that is ultimately used for rewarding research quality. Despite wide concerns about its harmful consequences to the UK higher education sector, the collective production of judgment makes the policy tool legitimate and undisputable as a mechanism enabling the allocation of finite financial resources. At the time of writing this paper, UK higher education institutions have received the result of the REF 2021 and university departments across the UK are already gearing up to the next round of evaluation.

Given the crucial importance of academic consensus for the legitimacy of the REF, it is striking how little seems to be understood about how consensus is achieved in fields best described as heterogeneous ‘machineries of knowing’ (Knorr Cetina, 1999) that are diverse and disunified, where knowing stems from esoteric and tacit interpretations. Searching for academic quality that is contingent to a specific epistemology and requires more time than the REF allows, how do academics agree to agree, and within constraints of a given timeframe? We argue that the situational character of academic quality is what enables a REF consensus. By looking closely at REF practices for achieving consensus on academic quality – a consensus which would seem to be impossible given the temporal and epistemological constraints – we show how academic quality is forged by the REF apparatus. To reach a consensus on academic quality is to set up a situation; but the terms of the situation become the terms of academic quality. Distinct from scientific or epistemic quality, the situational quality produced by the REF is what Jasanoff (2011, p. 33) calls the ‘serviceable truth’ of advisory (or regulatory) science. The REF translation of knowledge into policy decisions is based on expert consensus on academic quality. The transition from a scientist (peer reviewer) to an expert (REF panellist) is achieved through a situation that pacifies time concerns and harmonizes epistemic differences. Time and epistemological adherence are no longer an issue. Through a set of rules and the reconciliation of results that follows the implementation of rules, a scientist becomes an expert; epistemic truth on academic quality becomes situational truth. This truth performs the policy and money is distributed to the universities, but the mute shift between epistemic assessment and situated evaluation is dissociated from the REF. Results are produced in which any prior discontent is silenced, and these results stand as academic quality.

## Notes

1. The importance of the REF for institutions of higher education is also reflected in extensive scholarship concerned with the technicalities and effects of the REF and universities’ and disciplines’ responses to it (see, e.g., Karlsson, 2017; Kellard & Śliwa, 2016; Murphy, 2017; Murphy & Sage, 2014; Ní Mhurchú et al., 2017; Pidd & Broadbent, 2015; Pinar & Unlu, 2020; Sayer, 2015). For an excellent review of studies on the effects of using metrics in research evaluation see De Rijcke et al. (2016); for research on the impact component of the REF see Watermeyer (2016, 2019), Watermeyer and Chubb (2019).

2. In 2008, 67 panels had had 1,000 members, whereas the REF 2014 was done by 36 panels made up of 1,157 panellists (RAE, 2009; REF, 2014a).

3. The term ‘disciplinary’ might be slightly misleading here, as epistemological disunity (a variety of epistemic communities) obtains within, and not just between, disciplines. In the context of our argument more precise terms are ‘epistemic’ or ‘sub-disciplinary’ objectivity.

4. In a similar way, in his discussion of the grant evaluations in the US, Brenneis (1994) talks about ‘the structured character’ of ‘situational participation’ of peers in their reviewing applications, though he is not concerned with the question of consensus as such. We are grateful to one of our reviewers for pointing out this paper.
